# Strong Invasive Mechanism of *Wedelia trilobata* via Growth and Physiological Traits under Nitrogen Stress Condition

**DOI:** 10.3390/plants13030355

**Published:** 2024-01-25

**Authors:** Zhi-Cong Dai, Fang-Li Kong, Yi-Fan Li, Riaz Ullah, Essam A. Ali, Farrukh Gul, Dao-Lin Du, Yi-Fan Zhang, Hui Jia, Shan-Shan Qi, Nisar Uddin, Irfan Ullah Khan

**Affiliations:** 1 School of Emergency Management, Jiangsu University, 301 Xuefu Road, Zhenjiang 212013, China; daizhicong@163.com (Z.-C.D.); ddl@ujs.edu.cn (D.-L.D.); 2 Institute of Environment and Ecology, School of the Environmental and Safety Engineering, Zhenjiang 212013, China; 15555684308@163.com (F.-L.K.); liyifan05030@163.com (Y.-F.L.); farahgul196@yahoo.com (F.G.); zhangyi9821@163.com (Y.-F.Z.); jiahuiboru@126.com (H.J.); 3 Jiangsu Collaborative Innovation Center of Technology and Material of Water Treatment, Suzhou University of Science and Technology, Suzhou 215009, China; 4 Jingjiang College, Jiangsu University, Zhenjiang 212018, China; 5 Department of Pharmacognosy, College of Pharmacy, King Saud University, Riyadh 11451, Saudi Arabia; rullah@ksu.edu.sa; 6 Department of Pharmaceutical Chemistry, College of Pharmacy, King Saud University, Riyadh 11451, Saudi Arabia; esali@ksu.edu.sa; 7 School of Agricultural Engineering, Jiangsu University, Zhenjiang 212013, China; 8 Biofuels Institute, School of Emergency Management, School of the Environment and Safety Engineering, Jiangsu University, Zhenjiang 212013, China; shallnr@yahoo.com

**Keywords:** physiological parameters, tolerance capability, chlorophyll content, plant growth

## Abstract

Nitrogen (N) is one of the most crucial elements for plant growth. However, a deficiency of N affects plant growth and development. *Wedelia trilobata* is a notorious invasive plant species that exhibits superior tolerance to adapt to environmental stresses. Yet, research on the growth and antioxidant defensive system of invasive *Wedelia* under low N stress, which could contribute to understanding invasion mechanisms, is still limited. Therefore, this study aims to investigate and compare the tolerance capability of invasive and native *Wedelia* under low and normal N conditions. Native and invasive *Wedelia* species were grown in normal and low-N conditions using a hydroponic nutrient solution for 8 weeks to assess the photosynthetic parameters, antioxidant activity, and localization of reactive oxygen species (ROS). The growth and biomass of *W. trilobata* were significantly (*p* < 0.05) higher than *W. chinensis* under low N. The leaves of *W. trilobata* resulted in a significant increase in chlorophyll a, chlorophyll b, and total chlorophyll content by 40.2, 56.2, and 46%, respectively, compared with *W. chinensis. W. trilobata* significantly enhanced antioxidant defense systems through catalase, peroxidase, and superoxide dismutase by 18.6%, 20%, and 36.3%, respectively, providing a positive response to oxidative stress caused by low N. The PCA analysis showed that *W. trilobata* was 95.3% correlated with physiological traits by Dim1 (79.1%) and Dim2 (16.3%). This study provides positive feedback on *W. trilobata* with respect to its comprehensive invasion mechanism to improve agricultural systems via eco-friendly approaches in N deficit conditions, thereby contributing to the reclamation of barren land.

## 1. Introduction

Macronutrients are essential for the growth and survival of many living organisms, including plants, animals, and microorganisms. Nitrogen (N) is one of the crucial elements for plant growth and development [1]. It is a primary component of chlorophyll, proteins, and nucleic acids, and is thus involved in photosynthesis, enzyme activity, and growth [2]. Nitrogen optimization and remobilization play a vital role in promoting agricultural sustainability [3]. According to Meng et al. [4], approximately one billion hectares of agricultural land has been protected by the enhancement of nitrogen use efficiency in poor nutrient conditions. The N requirement for plants varies based on the type of plants and their growth stage; therefore, it is recommended to test the soil N level and fertilize [5]. Plants’ roots uptake N from the soil in the form of nitrate (NO_3_^−^) and ammonium ion (NH_4_^+^) [6]. The absorption of these two types of ions occurs via a membrane transporter located inside the roots, and it is then translocated to the other parts of the plants [7]. Basically, the ammonium transporter (AMT) and nitrate transporter (NRT) are responsible for adapting the N levels in plants. Under normal N conditions, plants exhibited better growth and physiological activities [8]. Excessive or low-N applications can lead to negative consequences, such as nutrient runoff, soil degradation, and altered ecosystem dynamics in agriculture [9,10].

Insufficient N levels in soil inhibit plant growth and secondary metabolites [11]. Generally, a lack of nitrogen obstructs enzymatic activities and increases reactive oxygen species (ROSs), leading to disturbed nutrient uptake activities in plants [12]. Low N reduces photosynthetic pigments, i.e., net fluorescence, stomatal conductance, chlorophyll content, and the assimilation of CO_2_ in plants [13]. The negative effects of low N were observed in rice, corn, pecan, and barley [8,14]. Physiological and biochemical processes are the main processes that respond to low-N environments via the roots and leaf tissues of plants [15]. The physiological exploration of plants involves the study of how plants acquire, transport, and use water, minerals, and other nutrients. It also includes the study of plant growth, development, photosynthesis, respiration, and senescence [16]. By understanding these processes, plant physiologists contribute to improving crop yields, developing disease-resistant varieties, and identifying ways to mitigate the effects of climate change on plant growth and development. Similarly, the biochemical exploration of plants focuses on the various biochemical pathways that occur within plants [17]. It involves the study of plant metabolism, which includes the synthesis and breakdown of carbohydrates, lipids, and proteins. It also includes the study of plant secondary metabolites, which are chemicals produced by plants that are not involved in primary metabolism under low-N conditions [18].

Different plant species have different responses to N deficiency, encompassing morphological, physiological, or biochemical ones [19]. Plants will undergo these changes to uptake more N in the soil to promote growth under low-N conditions [20,21]. Plant roots have the capability to adapt to different environmental stress conditions, especially under low-N conditions [22]. Besides, root morphology, leaf characteristics, and leaf area are also used as indicators against low-N conditions because chlorophyll content is directly connected with N availability [23]. Qiu et al. [24] reported that N content is directly proportional to photosynthesis, which is associated with chlorophyll content. *Moso bombo* is one of the woody plants that improved its morphological and physiological response to stress conditions under ammonium ion (NH_4_*)* [25]. Chen et al. [26] reported that *Populus deltoides* significantly promoted root growth that uptakes more N from the soil to improve nitrogen use efficiency under low-N conditions. Invasive plant species also have the capability to live in stressful conditions without any damaging effects because they have strong ecological adaptability [27]. *Sitaria italica* (L.) is one of the grassy plants that increased its root elongation, physiological response, and antioxidant defense system under low-N conditions [9]. *Solidago canadensis* enhanced its stem length, root length, and photosynthesis rate under different N conditions [28].

*Wedelia trilobata* (synonymous with *Sphagneticola trilobata*) is one of the native plant species of southwest America belonging to the family Asteraceae and was introduced to China as an ornamental base in the 20th century. However, this species has been accepted as an invasive plant in China, because it occupies more humid and subtropical areas [29]. *W. trilobata* is distributed throughout China, including the Guangxi, Hunan, Fujian, and Guangdong provinces [30]. *W. trilobata* has a well-developed root system, rapid growth, high nutrient uptake capability, and a strong antioxidant defense system in low-nutrient-availability conditions [31]. Invasive plant species were reported to have strong N adaptability to regulate the toxicity of heavy metals [32]. The responses of *W. trilobata* have proved to be positive in different environmental stress conditions. For example, its antioxidant activities and gene expression levels were higher compared with native *Wedelia* under drought stress [33]. The photosynthetic and physiological response of *W. trilobata* was significantly higher under low-temperature and -light conditions [30]. Previous studies observed that *W. trilobata* has a strong response to N enrichment and flooding conditions and better antioxidant enzyme activities under different forms of nitrogen compared with native *Wedelia* [34,35]. Until now, there has been no specific information on the physiological and biochemical application of *W. trilobata* under N deficit conditions. So, it is important to understand how *W. trilobata* responds to low N and how this response might be positive for agricultural systems. For this purpose, the aim of the current study was to investigate the comparison between *W. trilobata* and *W. chinensis* under N deficient condition and to evaluate its physiological, biochemical, and ecological adaptive invasion mechanism under normal and low-N conditions. This study will provide an in-depth and reliable approach for assessing invasive *W. trilobata* low-N adaptation, as well as the theoretical underpinnings for improving N application on various low-N-tolerant crops in barren lands. Future studies will be required to evaluate the molecular mechanism involved under low-N conditions.

## 2. Results

### 2.1. Comparison of Plants by Phenotypical and Growth Traits

**Figure 1:**
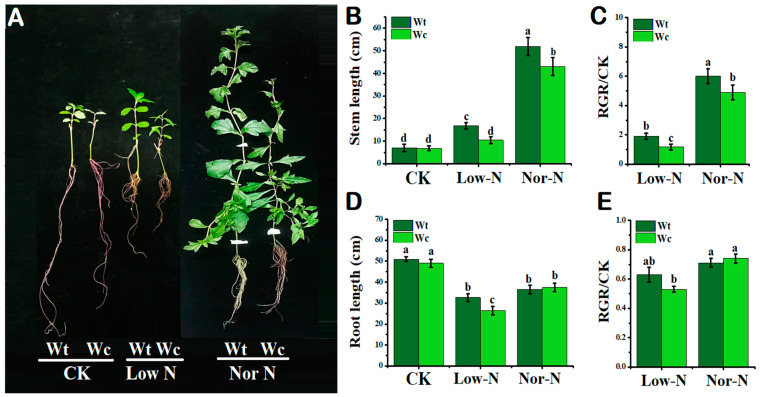
Growth response of *W. trilobata* (Wt) and *W. chinensis* (Wc). Sixteen-day-old plants with or without Hoagland nutrient solution supplied with low concentration of nitrogen (Low-N) and normal concentration of nitrogen (Nor-N) for 8 weeks: (**A**) Phenotypes of both species. (**B**,**C**) Stem length and RGR rate. (**D**,**E**) Root length and RGR rate with control, Low-N and Nor-N, respectively. Vertical bars indicate standard deviation in 6 replicates. The different letters represent significant differences in *W. trilobata* and *W. chinensis* with ANOVA (Tukey’s test, *p* < 0.05) analysis.

The growth phenotypes of native and invasive *Wedelia* were not significantly different under control conditions. Under the normal (Nor-N) and low-N (Low-N) Hoagland nutrient conditions, the growth phenotypes of *W. trilobata* were better compared with *W. chinensis* (Figure 1A). The stem length of the invasive *Wedelia* was significantly (*p* < 0.05) higher than native *Wedelia* by 17–38%, respectively, under Nor-N and Low-N conditions (Figure 1B). The relative growth rate (RGR) of the *W. trilobata* was higher than the control and *W. chinensis* under both levels of N conditions (Figure 1C). The root elongation was not significant from each other under the Nor-N condition; however, the root elongation of *W. trilobata* was higher than *W. chinensis* under Low-N conditions (Figure 1D). The number of roots, roots biomass, and root surface area of *W. trilobata* were significantly (*p* < 0.05) increased under Low-N conditions compare with *W. chinensis* (Table 1). This result suggests that *W. trilobata* has the capability to adjust growths in low-nutrient environments.

**Figure 2:**
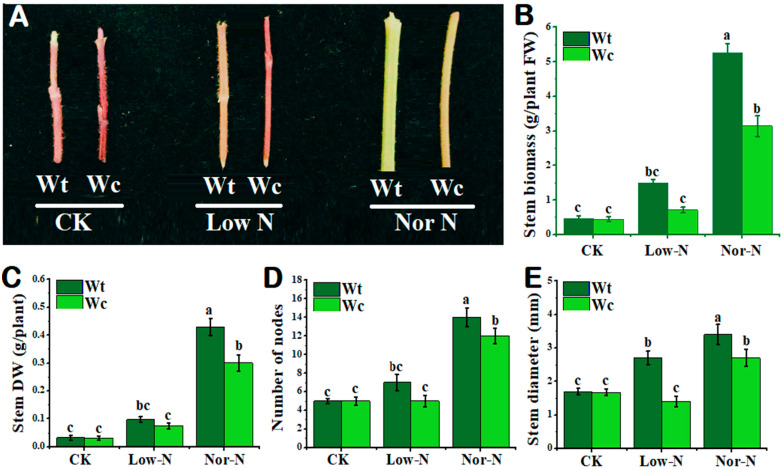
Growth response of *W. trilobata* (Wt) and *W. chinensis* (Wc)**.** Sixteen-day-old plants with or without Hoagland nutrient solution supplied with Low-N and Nor-N for 8 weeks: (**A**) Stem phenotypes of both species. (**B**) Stem biomass. (**C**) Stem dry weight. (**D**) Number of nodes and (**E**) stem diameter with control, Low-N, and Nor-N, respectively. Vertical bars indicate standard deviation in 6 replicates. The different letters represent significant differences in *W. trilobata* and *W. chinensis* with ANOVA (Tukey’s test, *p* < 0.05) analysis.

As shown in Figure 2A, the tolerance capability of *W. trilobata* was higher compared with *W. chinensis* under both levels of N conditions. The phenotypes of the invasive plant were stouter than native plants under Nor-N and Low-N conditions. The stem biomass (fresh and dry) of invasive *Wedelia* was significantly (*p* < 0.05) increased compared with native *Wedelia* under Nor-N conditions (Figure 2B,C). Similarly, the number of nodes in invasive *Wedelia* increased significantly compared with the native *Wedelia*, especially under Nor-N conditions (Figure 2D). The stem diameter of *W. trilobata* was greater by 48.4–20.5% than *W. chinensis* under Nor-N and Low-N conditions (Figure 2E).

### 2.2. Tolerance of Leaves under Different N Conditions

**Figure 3:**
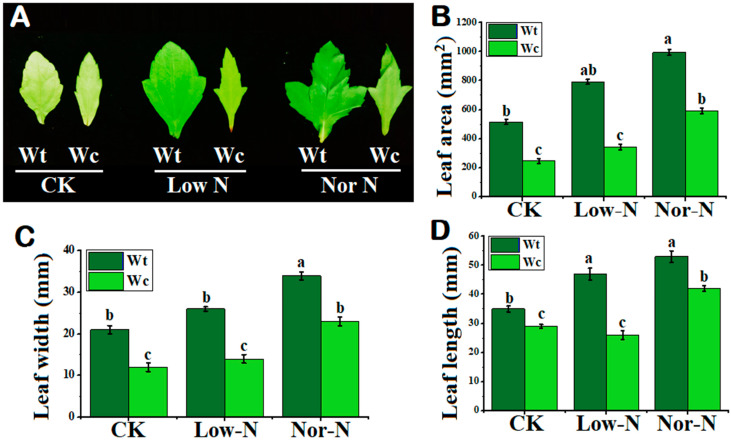
Leaf morphology of (Wt) *W. trilobata* and (Wc) *W. chinensis***.** Sixteen-day-old plants with or without Hoagland nutrient solution supplied with Low-N and Nor-N for 8 weeks: (**A**) Leaf phenotypes of both species. (**B**) Leaf area. (**C**) Leaf width. (**D**) Leaf length with control, Low-N, and Nor-N, respectively. Vertical bars indicate standard deviation in 6 replicates. The different letters represent significant differences in *W. trilobata* and *W. chinensis* with ANOVA (Tukey’s test, *p* < 0.05) analysis.

The leaves of *W. trilobata* were observed to be broader compared with native *Wedelia* under both levels of N conditions. The leaves of *W. trilobata* were greener compared with *W. chinensis*, especially under Low-N conditions (Figure 3A). The leaf area of *W. trilobata* was greater under normal and Low-N levels compared with native *Wedelia* (Figure 3B). Leaf width and leaf length were also smaller in the native species. Compared with native *Wedelia*, invasive *Wedelia* has a greater response to deficit N by increasing leaf width and length by 44.6% and 46.15%, respectively (Figure 3C,D). The number of leaves of *W. trilobata* was significantly enhanced under the N conditions compared with *W. chinensis* (Table 1). This suggested that the invasive *Wedelia* had a strong tolerance capability to adjust its own characteristics under Low-N conditions.

### 2.3. Leaf Photosynthetic Response to N Conditions

**Figure 4:**
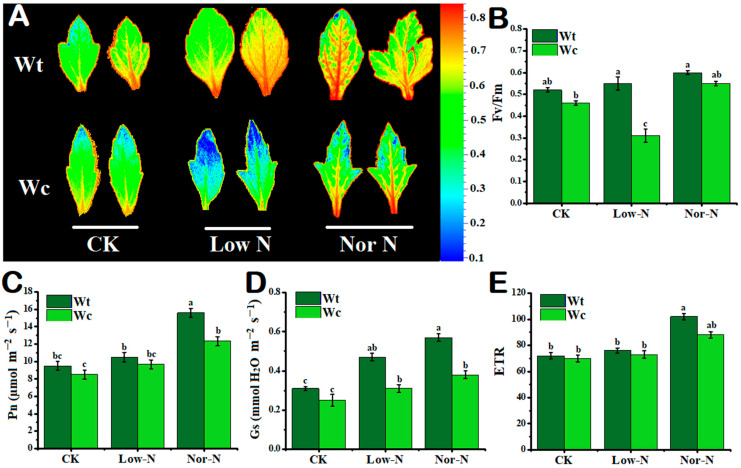
Growth response of (Wt) *W. trilobata* and (Wc) *W. chinensis***.** Sixteen-day-old plants with or without Hoagland nutrient solution supplied with Low-N and Nor-N for 8 weeks: (**A**) Morphology of chlorophyll fluorescence of both species. (**B**) *Fv/Fm*. (**C**) net photosynthetic rate. (**D**) Stomatal conductance and (**E**) electron transport rate with control, Low-N, and Nor-N, respectively. Vertical bars indicate standard deviation in 6 replicates. The different letters represent significant differences in *W. trilobata* and *W. chinensis* with ANOVA (Tukey’s test, *p* < 0.05) analysis.

The photosynthetic traits responses of *W. trilobata* were observed to be stronger than native *W. chinensis* under Low-N conditions. The chlorophyll fluorescence image clearly indicated that *W. chinensis* leaves changed from green color to blue, indicating toxicity under Low-N conditions; however, the invasive *Wedelia* has a strong tolerance to Low-N conditions (Figure 4A). There was no obvious effect observed in Fv/Fm (net fluorescence) under control and Nor-N conditions in both plant species; whereas, under Low-N conditions, the Fv/Fm value was significantly reduced in *W. chinensis* by 54.4% compared with the control, but *W. trilobata* does not affect net fluorescence levels (Figure 4B). The net photosynthetic rate was slightly increased in *W. trilobata* compared with *W. chinensis* with Nor-N (Figure 4C). The stomatal conductance (Gs) was significantly enhanced by 33.9% under Nor-N conditions in *W. trilobata* compared with *W. chinensis* (Figure 4D). The electron transport rate (ETR) did not differ significantly between the two species under both N conditions (Figure 4E).

**Figure 5:**
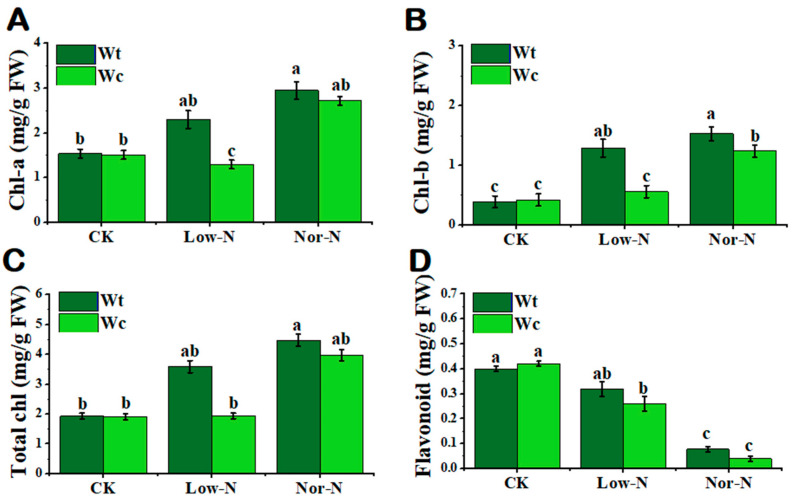
Physiological response of (Wt) *W. trilobata* and (Wc) *W. chinensis***.** Sixteen-day-old plants with or without Hoagland nutrient solution supplied with Low-N and Nor-N for 8 weeks: (**A**) chlorophyll-a. (**B**) chlorophyll-b and (**C**) total chlorophyll content of both plant species. (**D**) Flavonoid content with control, Low-N, and Nor-N, respectively. Vertical bars indicate standard deviation in 6 replicates. The different letters represent significant differences (*p* < 0.05) in *W. trilobata* and *W. chinensis* with ANOVA (Tukey’s test, *p* < 0.05) analysis.

Chlorophyll is another physiological indicator that plants respond to abiotic stress. In this study, the chlorophyll content was significantly inhibited in *W. chinensis* under Low-N conditions compared with *W. trilobata.* The chlorophyll-a and b content of *W. trilobata* was significantly increased by 40.2 and 56.2%, respectively, compared with *W. chinensis* under Low-N conditions (Figure 5A–C). The nonenzymatic antioxidant flavonoid content of *W. trilobata* was not significantly enhanced compared with *W. chinensis* under Low-N conditions (Figure 5D). There was no significant variation in total nitrogen content between both *Wedelia* species in the leaves; however, the nitrogen content of *W trilobata* was higher under Low-N and Nor-N conditions in the roots (Table 1).

### 2.4. Detection of Reactive Oxygen Species and Antioxidant Enzyme Activities of Plants in N Conditions

**Figure 6:**
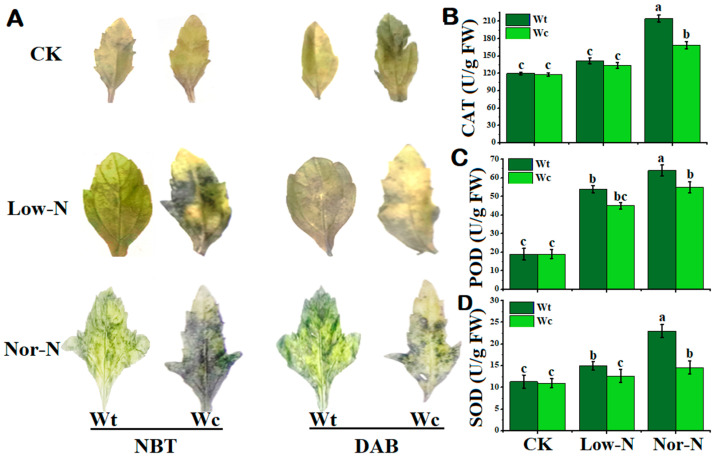
The accumulation of reactive oxygen species (ROSs) in the leaves and stems of *W. trilobata* and *W. chinensis* species under low and normal nitrogen conditions: (**A**) The changes in superoxide anion (O_2_^−^) and changes in hydrogen peroxide (H_2_O_2_) in the leaves of the two species after NBT and DAB staining. (**B**) Catalase activity. (**C**) Peroxidase activity and (**D**) superoxide dismutase activity of *W. trilobata* and *W. chinensis*. Vertical bars indicate standard deviation in 6 replicates. The different letters represent significant differences (*p* < 0.05) in *W. trilobata* and *W. chinensis* with ANOVA (Tukey’s test, *p* < 0.05) analysis.

To check the toxicity of *W. trilobata* and *W. chinensis* under deficit N conditions, this study used DAB and NBT staining to check for spots in the leaves. The stained spots were observed in both *Wedelia* plant species under N conditions. The leaves of *W. chinensis* exhibited a considerable number of deep-colored indigo spots (NBT) and brown spots (DAB) under Low- and Nor-N conditions, showing that more O_2_^−^ and H_2_O_2_ accumulated. *W. trilobata*’s stained spots were noticeably fewer than *W. chinensis*, indicating decreased accumulation of ROS and hydrogen peroxide (Figure 6A). Different trends of antioxidants were observed at different levels of N conditions in both plant species. The catalase (CAT) activity of both *Wedelia* plant species was not more significant than the control under Low-N conditions, while the CAT activity of *W. trilobata* was significantly increased by 66.9% compared with the control and 21.3% compared with *W. chinensis* under Nor-N conditions (Figure 6B). The peroxidase (POD) activity of both *Wedelia* species was increased compared with the control. Interestingly, the POD levels of *W. trilobata* were significantly higher by 16.3% under Nor-N conditions compared with *W. chinensis* (Figure 6C). The superoxide dismutase (SOD) trend of *W. trilobata* was significantly higher than the control, while the activity of *W. chinensis* was comparable to the control. The SOD activities of *W. trilobata* increased by 36.3% and 109%, respectively, compared with control under both levels of N conditions (Figure 6D). The result suggests that *W. trilobata* has a positive response to Low-N conditions, including scavenging ROS by antioxidant enzymes.

### 2.5. Correlation of Growth Traits and Physiological Activity

**Figure 7:**
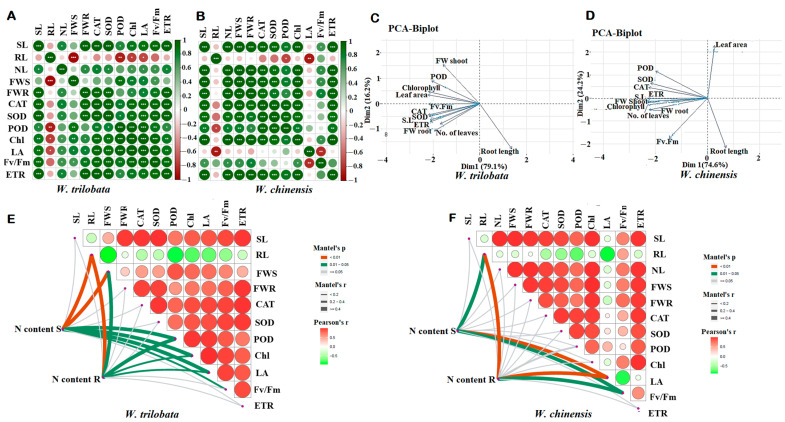
Pairwise correlation was used to identify the positive and negative interaction between the growth traits, i.e., shoot length (SL), root length (RL), number of leaves (NL), fresh weight shoot biomass (FWS), fresh weight root biomass (FWR), and CAT, POD, SOD, chlorophyll (Chl), leaf area (LA), photosystem (Fv/Fm), and the electron transport rate (ETR) of *W. trilobata* (Wt) and *W. chinensis* (Wc). Different asterisks show different correlations with each trait: (**A**) *W. trilobata*. (**B**) *W. chinensis*. Single, double, and triple asterisks show significant correlation levels. Similarly, principal component and Mantel test analysis revealed that different physiological parameters correlated with different growth traits: (**C**,**E**) *W. trilobata* and (**D**,**F**) *W. chinensis*.

A pairwise correlation was used to identify the positive and negative interaction between the growth traits, i.e., shoot length (SL), root length (RL), number of leaves (NL), fresh weight shoot biomass (FWS), fresh weight root biomass (FWR) and physiological activities such as CAT, POD, SOD, chlorophyll (Chl), leaf area (LA), photosystem (Fv/Fm), and the electron transport rate (ETR) of *W. trilobata* and *W. chinensis* (Figure 7A,B). Shoot length, fresh biomass, and number of leaves were strongly positively correlated with physiological indicators in both plant species; however, the root lengths of both plant species were negatively correlated with other traits under different nitrogen conditions. Biplot principal component analysis indicated different variations between the growth traits and physiological traits among the *W. trilobata* and *W. chinensis*. The biplot PCA analysis showed that the *W. trilobata* 95.3% correlated with Dim1 (79.1%) and Dim2 (16.3%), respectively (Figure 7C). Similarly, The PCA biplot correlation of *W. chinensis* also showed significant a correlation between the growth and physiological traits under different N conditions. PCA biplot analysis showed that both plant species were correlated positively and negatively for different traits (Figure 7C,D). Furthermore, the Pearson correlation was verified by the Mantel test for both *Wedelia* species for N content with other physiological and biochemical traits. The Mantel test analysis of *W. trilobata* showed that the N content in shoots and roots was highly significant (*p* < 0.01) with plant growth traits and other physio-bio traits; however, *W. chinensis* showed less significant correlation of growth traits and other traits compared with *W. trilobata* (Figure 7E,F).

## 3. Discussion

This study showed that the invasive plant species adapted its growth to environmental stressors, with green leaves being the principal photosynthetic part that responded to stressors. Similarly, the stem is a nonphotosynthetic organ that plays an important role in the invasion process. The current study used invasive plant species (*W. trilobata*) and native plant species (*W. chinensis*) cultured in hydroponic nutrient solution to investigate responses to low-N conditions. This study investigated the comparison between native and invasive plant species under low and normal N conditions. Moreover, we also revealed the invasion mechanism of *W. trilobata* and its antioxidant defense system under low-N conditions. The current study helps us to truly understand the contribution of plants’ own physiological and ecological advantages to successful invasion.

### 3.1. The Growth Response of W. trilobata Is Better Than W. chinensis under Low-N Conditions

Generally, plant growth and development require all the essential micro- and macronutrients in normal conditions [36]. Nitrogen is one of the major macronutrients that plays an important role in plants, such as the formation of proteins, DNA preparation, chlorophyll, and plant hormones [22]. When the concentration of N is insufficient, the plant will decrease its growth, development, and other life activities [23]. A deficiency of N affects plant height, root length, leaf traits, stem width, biomass, and other growth factors [37]. Different plant species have different effects and responses to low-nutrient conditions, such as modifying root architecture, enhancing N assimilation, leaf photosynthetic responses, lignin, chloroplast, and carbohydrate metallization [38,39,40]. Similarly, native and invasive plant species are also affected under low-N environment conditions [27]. This study observed a positive response in *W. trilobata* to low-N conditions, namely via root elongation, an increase in the number of roots, leaf morphology, and increased leaf numbers and stem width. However, the root length growth of native *Wedelia* was inhibited, as well as stem length and leaf structure, under low-N conditions. This result suggests that the invasive *Wedelia* has a positive response to low N because it absorbs and stores more nutrients and organic compounds through roots to adjust its life activities, including changing the resource allocation strategies under poor nutrient conditions to cope with adverse habitats. In N deficit conditions, this essential nutrient is transported to the upper part of the plant via the xylem transporter, which provides a normal situation [41]. Foxtail millet developed specific root lengths and diameters to adapt to the growth under low-N conditions that are similar to the findings of the current study [9]. It has already been proved that invasive plant species have great responses and stronger competitive capabilities under low-nutrient environments by improving their regulatory mechanisms, as well as better growth adjustment compared with native plant species [40,42,43].

The leaf is the main plant organ that is directly linked to nitrogen content and provides positive responses to low-nutrient environments [44,45]. The leaf morphology of *W. trilobata* was larger and thinner to increase the specific leaf area that enhanced its tolerance under low-N conditions. In contrast, in native *Wedelia*, the size of its leaf is thicker and softer due to decreased leaf-specific area under low-N conditions compared with invasive plants and the control. Likewise, the leaf width, leaf area, leaf length, and number of leaves increased compared with native *W. chinensis* under both levels of N conditions because t has more absorption and transport activities of N from the soils and adapted quickly to the environments [46]. Cai et al. [47] revealed that the invasive *W. trilobata* leaves are denser, larger, and wider compared with native *S. trilobata* due to the capability of the accumulated essential hormones (auxin and cytokinin) to increase the specific leaf area under poor nutrient conditions. The leaves of *W. trilobata* were much improved compared with native *W. chinensis* after the amendments of different forms of N such as nitrate ion and ammonium ion (NO_3_^−^, NH_4_^−^), both alone and in mixed forms [27]. This study concluded that the invasive *Wedelia* has a strong capability to adjust its life characteristics under different stressful conditions, such as low-N conditions, through invasion mechanisms compared with native plant species.

### 3.2. Physiological Response of W. trilobata to Low-N Conditions

Photosynthesis and chlorophyll are the main physiological indicators of plant responses to abiotic stress [36]. Invasive plant species have strong photosynthetic capacity compared with native plant species. For example, the invasive *Wedelia* had greater net photosynthetic pigment, stomatal conductance, transpiration rate, and gas exchange capacity compared with native *Wedelia* under different environmental conditions [30]. According to the current study, the photosynthetic trait responses of *W. trilobata* were observed to be stronger than native *W. chinensis* under low-N conditions. The chlorophyll fluorescence image clearly indicated that *W. chinensis* leaves change from green color to blue, showing toxicity under low-N conditions; however, the invasive *Wedelia* has a strong tolerance to low-N conditions because the leaf size and area are wide, storing more essential nutrients by chlorophyll. Similarly, the Fv/Fm value was greater than *W. chinensis* under low-N conditions. Similar results were also reported by Ke et al. [35]. *W. trilobata* have stronger net fluorescence Fv/Fm than *W. chinensis* under low-light conditions, demonstrating that low-light conditions accelerate the photosystem II reaction that converts light energy to heat energy. Net photochemical efficiency (Fv/Fm) is commonly used for the detection of fluorescence traits, with the normal range being from 0.7 to 0.8 and significantly decreased under stressful conditions [48]. The present study also observed that *W. trilobata* increased its stomatal conductance and net transpiration rate under the Nor-N conditions (Figure 4). Consistent with the current study, Cai et al. [47] indicated that *S. trilobata* decreases its trend of Fv/Fm and stomatal conductance under low-temperature conditions compared with native plant species. This result suggests that when contrasted with extant native plants, *W. trilobata* exhibits greater invasion, allelopathy, and phenotypic adaptability attributes that allow it to quickly adapt and colonize under low-N conditions.

Nitrogen content directly influences the net photosynthesis of plants through chlorophyll formation, which regulates a particular photosynthetic enzyme in plant tissues, as well as chloroplast quantity, size, and composition [49]. Under low-N conditions, the plant’s chloroplast size, quantity, and composition decreased due to the decreased expression levels of photosynthetic genes [39]. This study also observed a decreasing trend in chlorophyll a, b, and total chlorophyll content under low-N conditions compared with the normal provision of N content, which may be due to the destruction of the chloroplast and photosynthetic organ. However, the chlorophyll a and b content of invasive *Wedelia* was significantly enhanced compared with native *Wedelia*, which means *W. trilobata* has strong photosynthetic pigments and chloroplast activities under N deficit conditions. Similar findings have been reported under low-temperature conditions [30,47]. The second reason that might cause *W. trilobata*’s response to low-N conditions is the decreased synthesis of specific organic compounds, such as amino acids, nucleic acids, proteins, and other essential compound that are required for good photosynthetic properties [50]. Switch grass (low-land-type) enhanced the chlorophyll content under low-N conditions because it has a strong capability to survive in poor nutrient conditions, which is consistent with the current study [51]. Overall, the results suggest that the invasive *W. trilobata* has a strong tolerance compared with native *W. chinensis* under different environmental stressors.

### 3.3. Alleviation of ROS by Defensive Antioxidant Enzyme Activities

Abiotic stressors lead to the generation of numerous reactive oxygen species (ROSs). Among these, O_2_^•−^ plays a pivotal role in plants, serving as the primary catalyst for the formation of other ROS [52]. Few reports indicated that the scavenging systems may be lost by the production of high amounts of ROS, and antioxidant enzyme activity can efficiently maintain balanced levels of ROS in plants [53,54,55]. The present study clearly indicated the accumulation of ROS with the help of DAB and NBT staining by observing the different spots in the leaves of *W. trilobata* and *W. chinensis* under low-N conditions. The current observation indicated that the invasive *Wedelia* had fewer spots than native *Wedelia*, which means *W. trilobata* activated antioxidant activities to maintain its levels of ROS under low-N conditions. Reactive oxygen molecules (ROMs), such as H_2_O_2_ and OH, accumulate in plants under stressful conditions and are harmful to crops because they interfere with their regular biological processes [31]. The H_2_O_2_ and O_2_^•−^ species activate various antioxidant enzyme defense systems in plants [39].

In order to absorb radicals and minimize ROS-mediated injury in plants, the concentrations of CAT, POD, and SOD are elevated. This study also checked the tolerance capability of invasive and native *Wedelia* to activate the antioxidant defensive systems under low-nutrient conditions. This study showed that the invasive *Wedelia* has a strong antioxidant system, including CAT, POD, and SOD, in poor nutrient conditions compared with native *Wedelia*. The CAT, POD, and SOD levels of *W. trilobata* were significantly higher compared with the control and native plant species. SOD is a catalyst for decreasing the cytotoxic conversion of O_2_^•−^ to H_2_O_2_, which may be crucial for plant cells to increase their antioxidant defense systems for tolerance [56]. When the antioxidant enzyme defensive system was activated, the production of H_2_O_2_ and ROS was catalyzed to help *W. trilobata* species resist environmental stress better than native plant species [47]. Farid et al. [57] reported that increasing the concentration of CAT and POD occurred when the plants were struggling to maintain their normal growth under environmental stress conditions. Similar to the current study, Cai et al. [30] reported that *S. trilobata* increased its flavonoid content, phenol content, and antioxidant defense systems in the stem/leaf compared with native plant species. Electrolyte leakage and membrane damage occur due to ROS accumulation, either directly or indirectly, to initiate membrane lipid peroxidation in plants [42]. Huang et al. [27] also reported *W. trilobata* to have activated strong antioxidant defense systems under the provision of excess nitrogen compared with *W. chinensis* to adapt itself by various chemical reactions. Plants produce a vast number of antioxidants, including antioxidant enzymes and nonenzymatic compounds, to eliminate too many ROS from their cells, for example, under drought stress conditions. The concentration of flavonoids and total phenols, as well as the activity of antioxidant enzymes (SOD, CAT, and POD), significantly increased in the leaves of the native and invasive plant species [22,42,58,59].

In summary, both plant species (*W. trilobata* and *W. chinensis*) were correlated positively and negatively with each other under low and normal N conditions in terms of shoot length, root length, biomass, and physiological parameters. The PCA biplot and Pearson correlation essay indicated that *W. trilobata* has strong physiological parameters to adjust its normal growth under low-N conditions compared with *W. chinensis*, which agrees with the idea of Iqbal et al. [60], who described the Pearson correlation and PCA analysis under different N conditions with different physiological parameters in different crops.

## 4. Materials and Methods

For this study, the invasive *W. trilobata* and indigenous *W. chinensis* plant species were obtained from Guangxi Province, Nanning City (22°38′ N and 108°13′ E) and the greenhouse of the Institute of Environment and Safety Engineering (32°12′2″ N, 119°30′50″ E) at Jiangsu University, respectively. The ramets of these two plant species were cut in two nodes and grown in the growth chamber till to two leaves were opened. For roots and germination, the stem segments were placed into an open, transparent glass jar in a chamber with an ambient light level of 95 mol m^−2^ s^−1^, a light cycle of 14/10 h, and an ambient temperature of 28 °C. After 16 d, the seedlings were transplanted in different experimental 500 mL plastic pots.

### 4.1. Experimental Design

Sixteen days later, nitrogen-free Hoagland nutrient solution was provided to each pot and a normal concentration of nitrogen (Nor-N) 91.05 mg/L and a low concentration of nitrogen (Low-N) 0.9105 mg/L were added in the form of eques calcium nitrate tetrahydrate (Ca(NO_3_)_2_·4(H_2_O)). The detailed research design included control CK (only water), low N (0.9105 mg/L with Hoagland nutrient), and normal N (Nor-N, 91.05 with Hoagland nutrient). The plants were grown in the treatments for 8 weeks with six replicates. Totally, 3 treatments × 2 species × 6 replicates equaled 36 pots, respectively (Table 2). Hoagland nutrient solution was renewed three times a week, and the pH levels were maintained at 5.8. The plastic pots’ openings were sealed with a hygienic air-filtering sheet.

### 4.2. Phenotypes and Growth Measurement

We used a DSLR digital camera (D7000 Camera Price-030I, Jiaxing factory, Jiaxing, city, Zhejiang, China.) for the phenotypical analysis of *W. trilobata* and *W. chinensis* at the end of the experiment. All parameters of each replicate of both plant species were taken and measured. We measured both plant species’ stem length, root height (cm), number of nodes, number of leaves, and number of branches. Roots, stems, and leaves were separated by seizer, and the fresh biomass was recorded in an experimental notebook [61]. Some fresh samples of both plant species were stored in liquid nitrogen at −80 °C for further analysis, and the remaining samples were kept in an oven for drying at 80 °C. WinRHIZO root analyzer system was used to analyze the root length, root volume, and number of root forks of *W. trilobata* and *W. chinensis* [38].

### 4.3. Analysis of Leaf Morphology

At the end of the experiment, new leaves of both plant species were cut in each treatment with 6 replicates. The leaf area (mm^2^) was estimated using a portable hand leaf area meter (Yaxin-1241, Shanghai, China). We used leaf image area software (ImageJ software, Version10.12.6), and leaf width was determined by Vernier caliper. After that, all leaf samples were placed in an oven for drying for 2–3 d to obtain biomass [46].

### 4.4. Analysis of Photosynthetic Parameters

Photosynthetic pigmentation was determined using a Fluor-Pen handheld chlorophyll fluorescence meter (Li Cor-USA Biosciences, Lincoln, NE, USA). This study used chlorophyll fluorescence image systems (CF Imager, Technological Ltd., Colchester, UK) to check F_v_/F_m_ values in dark and light conditions. For 20 min, the leaves were left in the dark. The dark-adapted leaves’ minimum fluorescence (Fo) and maximum fluorescence (Fm) were measured. The minimum fluorescence (F_0_) after 15 m in a light environment and maximum fluorescence (Fm) were measured after dark with the formula F_v_/F_m_ = (F_m_ − F_o_/F_m_) in control and treatments of the *W. trilobata* and *W. chinensis* [34]. A CIRAS-3 portable photosynthesis system (PP Systems, Amesbury, MA, USA) was used to measure the net photosynthetic rate (Pn), transpiration rate (ETR), and stomatal conductance (gs) of *W. trilobata* and *W. chinensis* [62]. Nitrogen content was analyzed with the protocol of Khan et al. [62].

For the analysis of chlorophyll content, fresh leaves of both *W. trilobata* and *W. chinensis* were crushed into small pieces, kept in 20 mL tubes, and stored in a dark place with the addition of 85% acetone to remove all the green color. The chlorophyll-a, chlorophyll-b, and total chlorophyll (mg/g^−1^ FW) content was checked with a spectrophotometer (UV-2550, Shimadzu, Kyoto, Japan) at 645 and 663 nm after 2 to 3 days according to Khan et al. [36].

Chlorophyll content (mg/g FW): Chl-a = 12.71 (OD663) − 2.59 (OD645); Chl-b = 22.88 (OD645) − 4.67 (OD663); Total Chl = Chl-a + Chl-b.

### 4.5. Measurement of Nonenzymatic Flavonoid Content

Flavonoid concentration was assessed using a colorimetric method on frozen plant tissues that were earlier preserved at −80 °C [47]. With the addition of 0.3 mL of 5% sodium nitrite, an aliquot of 1 mL of the extract was diluted with 4 mL of distilled water. Then, 10% AlCl_3_ was added to the solution after 5 min. After 6 min, 2 mL of 1 M NaOH was added, and 10 mL of distilled water was added to make the sample volume. An EON microplate spectrophotometer (Bio Tek, Vermont, VT, USA) was used to measure the absorbance at 510 nm after the solution was thoroughly mixed.

### 4.6. Localization of ROS with NBT and DAB Solution

The full leaves of *W. trilobata* and *W. chinensis* with control and treatment were immersed in diaminobenzidine (DAB) solution containing 0.5 mg/mL^−1^ with pH 8.0. Phosphate buffer was used as the solvent for 15 min before being covered with gauze and kept in the dark for 7 h. The accumulation of ROS could be seen as brown dots after DAB staining [35]. Similarly, both plant species were immersed in a solution using 0.1% nitro-blue tetrazolium (NBT) and 10 mM sodium azide using pH 6.4 phosphate buffer as the solvent. After being vacuum-covered for 20 min, the gauze was left in complete darkness for 2–3 h. The greenery in the leaves was removed by boiling 90% ethanol solution. Blue specks were observed after staining.

### 4.7. Analysis of Antioxidant Enzyme Activity

The antioxidant enzyme activity was assessed following the protocol outlined by Khattak et al. [63] with slight modification. Three (3) mg fresh leaf samples of *W. trilobata* and *W. chinensis* were collected and homogenized thoroughly in ice and crushed in liquid nitrogen. After crushing, a 4 mL phosphate buffer (PBS) (0.05 M Na_2_HPO_4_ + 0.05 M NaH_2_PO_4_) with pH 6 to 7 was added. The obtained samples were centrifuged at 12,000 rpm for 10 min at 4 °C. The supernatants were placed in ice for analysis of antioxidant enzyme activity.

The 3 mL enzymatic reaction mixture contains 1.9 mL of PBS (50 mM, pH 7), 0.1 mL of enzyme extract, and 1 mL of hydrogen peroxide (0.3%), and 0.2% of guaiacol with PBS was used as blank control. The peroxidase (POD) activity was determined by visible spectrophotometer at 470 nm [64]. The enzymatic reaction mixture for catalyzing (CAT) activity consists of 1 mL of 0.3% H_2_O_2_, 1.9 mL of H_2_O, and 0.1 mL of enzyme extract solution, with distilled water used as blank control. The CAT activity was determined by spectrophotometrically at 240 nm, and the enzyme was recorded every 30 s. At least 6 points were measured. The CAT was measured with the following formula, CAT = (△OD_240_ × V)/(0.01 × V_S_ × m), where m is the fresh biomass of the sample, vs. is the enzyme extract, and V is the total volume of the samples. The SOD and CAT values are expressed in U/g FW.

### 4.8. Statistical Analysis of the Study

All value parameters of *W. trilobata* and *W. chinensis* were put in Microsoft Excel software 2010. This study exemplifies experimental design, with six replicates for each treatment and species, allowing for a comprehensive representation of standard error. ANOVA, along with the Tukey *t*-test (*p* < 0.05), was conducted to derive meaningful statistical analysis from the data. The different letters indicate significant differences in all physicochemical and biochemical analyses. Correlation arrays were made by the heatmap to examine relationships between physicochemical and plant growth parameters. For the purpose of calculating PCA bilateral correlation factors between the variables, we used the “Performance Analytics” package, which uses the Pearson method. The Mantel test analysis was analyzed between the two matrices. The larger the correlation coefficient of the Mantel test, the smaller the *p*-value of both *Wedelia* species by online correlation tool (https://www.cloudtutu.com/#/index, accessed on 2 December 2023).

## 5. Conclusions

In conclusion, this study clearly indicated that the invasive *Wedelia trilobata* has a strong response to low-N conditions by improving plant height, stem length, leaf morphology, and root length. The physiological parameters, such as net photosynthesis, stomatal conductance, chlorophyll content, and flavonoid content, were significantly increased, showing a tolerance capability against low-N conditions. Under low-N conditions, the *W. trilobata* activated its self-enzymatic defensive mechanism to adjust its growth characteristic by catalase, peroxidase, and superoxide dismutase to resist the poor nutrient conditions. The NBT and DAB staining clearly show that *W. trilobata* has a strong response to low-N conditions compared with native *W. chinensis*. From the result of this study, it is concluded that *W. trilobata* could be a valuable option for ecosystem restoration and land management efforts. Additionally, it has the potential to contribute to environmentally friendly approaches, promoting ecological balance and reducing the negative impacts of low nitrogen. Further study will be required to explore the full potential of *W. trilobata* in environmental management and interactions with other plants, as well as its role in ecosystem restoration.

## Figures and Tables

**Table 1:** Growth parameter of *W. chinensis* and *W. trilobata* under Low-N and Nor-N conditions.

Treatments	CK		Low-N		Nor-N	
Plant Species	Wt	Wc	Wt	Wc	Wt	Wc
**No of leaves**	8 ^d^	8 ^d^	12 ^b^	10 ^c^	53 ^a^	48 ^ab^
**FW of leaves (g)**	0.28 ^e^	0.26 ^e^	0.63 ^c^	0.56 ^d^	8.8 ^a^	6.9 ^b^
**DW of leaves (g)**	0.07 ^e^	0.025 ^e^	0.085 ^c^	0.06 ^d^	0.87 ^a^	0.6 ^b^
**No. of roots**	7 ^c^	8 ^c^	13 a	10 ^b^	15 ^a^	14 ^a^
**FW of roots (g)**	0.34 ^b^	0.33 ^b^	0.27 ^b^	0.2 ^c^	1.7 ^a^	1.36 ^ab^
**DW of roots (g)**	0.031 ^b^	0.03 ^b^	0.031 ^b^	0.025 ^c^	0.1 ^a^	0.09 ^a^
**Root surface area (cm^2^)**	16 ^d^	15 ^d^	25 ^c^	15 ^d^	42 ^a^	35 ^b^
**N content in leaves (mg/g)**	4.66 ^b^	4 ^b^	7.33 ^ab^	5.33 ^b^	11.33 ^a^	10.67 ^a^
**N content in roots (mg/g)**	6 ^d^	5 ^d^	10 ^c^	7 ^d^	13.6 ^a^	11 ^b^

Wt: *W. trilobata* Wc: *W. chinensis*. Within the group, different letters indicate significant differences from each other (Tukey’s test, *p* < 0.05).

**Table 2:** The table indicates different concentrations of N used in the present study.

Serial No.	Treatments	CaNO_3_ (mg kg^−1^)
1	Wt-CK	0 (only water)
2	Wc-CK	0 (only water)
3	Wt-Nor-N	91.05 (with Hoagland)
4	Wc-Nor-N	91.05 (with Hoagland)
5	Wt-Low-N	0.9105 (with Hoagland)
6	Wc-Low-N	0.9105 (with Hoagland)

Wt (*W. trilobata*); Wc (*W. chinensis*); Nor-N (normal nitrogen); Low-N (low nitrogen).

## Data Availability

The data presented in this study are available on request from the corresponding author (e-mail: irfanullahkhan195@yahoo.com). The data are not publicly available due to some part of ongoing research to keep it confidential until further publications or patents are completed.
